# The impact of urbanization and population density on childhood *Plasmodium falciparum* parasite prevalence rates in Africa

**DOI:** 10.1186/s12936-017-1694-2

**Published:** 2017-01-26

**Authors:** Caroline W. Kabaria, Marius Gilbert, Abdisalan M. Noor, Robert W. Snow, Catherine Linard

**Affiliations:** 1 African Population and Health Research Centre, Nairobi, Kenya; 2 Spatial Health Metrics Group, Kenya Medical Research Institute/Wellcome Trust Research Programme, Nairobi, Kenya; 3 Spatial Epidemiology Laboratory, Université Libre de Bruxelles, CP160/12, Av. F.D. Roosevelt 50, 1050 Brussels, Belgium; 4 Fonds National de la Recherche Scientifique (F.R.S-FNRS), Brussels, Belgium; 5 Centre for Tropical Medicine and Global Health, Nuffield Department of Clinical Medicine, University of Oxford, Oxford, UK; 6 Department of Geography, Université de Namur, Rue de Bruxelles 61, 5000 Namur, Belgium

**Keywords:** Malaria, Urbanization, Population density, Boosted regression trees

## Abstract

**Background:**

Although malaria has been traditionally regarded as less of a problem in urban areas compared to neighbouring rural areas, the risk of malaria infection continues to exist in densely populated, urban areas of Africa. Despite the recognition that urbanization influences the epidemiology of malaria, there is little consensus on urbanization relevant for malaria parasite mapping. Previous studies examining the relationship between urbanization and malaria transmission have used products defining urbanization at global/continental scales developed in the early 2000s, that overestimate actual urban extents while the population estimates are over 15 years old and estimated at administrative unit level.

**Methods and results:**

This study sought to discriminate an urbanization definition that is most relevant for malaria parasite mapping using individual level malaria infection data obtained from nationally representative household-based surveys. Boosted regression tree (BRT) modelling was used to determine the effect of urbanization on malaria transmission and if this effect varied with urbanization definition. In addition, the most recent high resolution population distribution data was used to determine whether population density had significant effect on malaria parasite prevalence and if so, could population density replace urban classifications in modelling malaria transmission patterns. The risk of malaria infection was shown to decline from rural areas through peri-urban settlements to urban central areas. Population density was found to be an important predictor of malaria risk. The final boosted regression trees (BRT) model with urbanization and population density gave the best model fit (Tukey test p value <0.05) compared to the models with urbanization only.

**Conclusion:**

Given the challenges in uniformly classifying urban areas across different countries, population density provides a reliable metric to adjust for the patterns of malaria risk in densely populated urban areas. Future malaria risk models can, therefore, be improved by including both population density and urbanization which have both been shown to have significant impact on malaria risk in this study.

**Electronic supplementary material:**

The online version of this article (doi:10.1186/s12936-017-1694-2) contains supplementary material, which is available to authorized users.

## Background

Although malaria has been traditionally regarded as less of a problem in urban areas compared to neighbouring rural areas, the risk of malaria infection continues to exist in densely populated, urban areas of Africa. The process of urbanization and accompanying demographic change is associated with decreased risks of infection due to reduction of suitable breeding grounds for malaria vectors through reduction of vegetative cover, water surfaces and other natural surfaces with building structures and other paved surfaces as well as through pollution of available breeding sites. The reduction of breeding sites reduces the number of malaria vectors as well as their species diversity with the dominant malaria vectors in Africa, *Anopheles gambiae* s.s. and *Anopheles funestus,* shown not to proliferate well in urban habitats [1–4]. Low entomological inoculation rates (EIR) have been linked to high human population densities in urban areas [5, 6].

Recent efforts to map the intensity of malaria transmission in Africa have used urbanization to “force down” infection risks [1, 7–12]. Omumbo and colleagues [13] used discriminant analysis to examine impact of urbanization in Kenya, Tanzania and Uganda using investigator-defined urban/rural assignments supplemented by the urbanization definition from the Global Rural Urban Mapping Project (GRUMP). The inclusion of urbanization was found to improve the consistency of predictive malaria prevalence maps when compared with expert opinion maps [12]. However, the effects of urbanization were difficult to define due to discrepancies in urban classification, the coarse spatial resolution of climate data and poor coverage of malaria training data in the study [11, 12]. Hay and colleagues using GRUMP urban extents refined using population density showed urbanization was associated with lower entomological inoculation rates (EIR) compared to peri-urban and rural areas. However, there were inherent uncertainties in translation of EIR into prevalence of malaria infection [1]. Tatem et al. [14] found that GRUMP urban extents (GRUMP-UE) produced the most accurate match to author-defined urbanization. GRUMP-UE were then combined with population density to discriminate between malaria-relevant urban and ‘peri-urban’. This definition of urbanization has been used in recent global maps of *Plasmodium falciparum* endemicity as a prior covariate in the space–time geo-statistical model used in predicting parasite prevalence [8–10].

Despite the recognition that urbanization influences the epidemiology of malaria, there is little consensus in defining urbanization for malaria parasite mapping. Large-scale spatial datasets of urbanization developed in the last two decades vary in terms of spatial and temporal resolution of input census data, satellite imagery and spatial population allocation methods which results in differing extents of urban areas [15]. Previous studies examining the relationship between urbanization and malaria transmission have used products defining urbanization at global/continental scales developed in the early 2000s, that overestimate actual urban extents while the population estimates are over 15 years old and estimated at administrative unit level [8–10].

Defining a consistent/accurate gradient between rural and urban settlements is an important factor when defining malaria transmission patterns and has an influence on the estimated impact of urbanization on malaria parasite prevalence. The transition from a rural settlement to one best described as urban does not follow definite boundary gradations but transitions gradually similar to malaria transmission patterns. This study therefore sought to discriminate an urbanization definition that is most relevant for malaria parasite mapping using individual level malaria infection data obtained from nationally representative household-based surveys. Boosted regression tree (BRT) modelling was used to determine the effect of urbanization on malaria transmission and if this effect varied with urbanization definition. In addition, the most recent high resolution population distribution data was used to determine whether population density had significant effect on malaria parasite prevalence and if so, could population density replace urban classifications in modelling malaria transmission patterns.

## Methods

### Malaria prevalence data

Malaria parasite prevalence data was assembled from national cluster sample household surveys: Demographic and Health Surveys (DHS) [16]; Malaria Indicator Survey (MIS) [17]. Data was assembled from 19 household surveys conducted between 2007 and 2013 in 14 malaria endemic countries across Africa where information on malaria infection prevalence and household geographic coordinates was available (Additional file 1: Table S1). For each child identified from the household survey data, information was also assembled on factors that might influence malaria prevalence at child, household and cluster levels from the household survey datasets. A cluster is a group of adjacent households that serves as the primary sampling unit and is often defined using enumeration areas (EAs) provided by the most recent national population census. The following child related variables were assembled from the survey data: child’s age in months, child’s gender, whether the child tested positive for *P. falciparum* and the malaria testing method used [microscopy or rapid diagnostic tests (RDT)]. Information was also linked on whether the child slept under an ITN the previous night; whether children who had been febrile in the last 2 weeks had received an anti-malarial drug in the previous 2 weeks. Variables related to the mother (caretaker) of the index child assembled included the mothers’ age and education level. Variables that related to the household where the child lived included the application of IRS during the previous year; the number of nets available in a household and household size. The household wealth index, a composite measure of household cumulative living standards by quintiles (poorest, very poor, middle, fourth, and highest) was also recorded.

Finally, data on extrinsic determinants of malaria transmission related to the cluster where the index child lived was assembled from remotely sensed environmental datasets. Geographic coordinates of each cluster were used to ascribe to every child-record annual mean temperature, temperature suitability index (TSI), precipitation, enhanced vegetation index (EVI), and malaria seasonality index. Two indicators of temperature were used in this study, annual mean temperature and temperature suitability index (TSI). Annual mean temperature was calculated from monthly mean temperature (1950–2000) available at 1 km resolution grids from WorldClim global climatology dataset [18]. TSI was developed from a biological model that accounts for the dependency of the malaria transmission cycle on temperature developed to provide a biologically relevant suitability index [19]. Information on rainfall was extracted from a synoptic annual mean precipitation grid calculated from monthly total precipitation gridded datasets obtained from WorldClim global climatology dataset. EVI values were extracted from Fourier-processed AVHRR data available at 1 km resolution gridded surface [20]. The influence of seasonal variation in malaria transmission was accounted for using a malaria seasonality index. The malaria seasonality index was derived from daily rainfall estimates from the African Rainfall Estimates version 2 (RFE 2.0) dataset developed as a collaborative programme between NOAA’s Climate Prediction centre (CPC) and USAID/Famine Early Systems Network (FEWS). Daily-accumulated rainfall data between 2002 and 2009 was used to identify areas that received 60% of annual rainfall within 3 months which was found to best fit seasonal clinical malaria profiles [21]. All extractions were done using the spatial analyst tool in ArcGIS 10 (ESRI, USA). The average value within 5 km of the cluster centre in rural areas and 2 km in urban areas was calculated to account for household dispersal with a cluster and additionally account for random positional error deliberately introduced for confidentiality purposes in DHS surveys reporting HIV by up to 5 km in rural areas and 2 km or less in urban areas [22].

### Urbanization classification

The urbanization status of the household was recorded during the DHS and MIS surveys, based on a national classification of urban sample clusters defined by national Central Statistics Offices (CSO). The definitions are often not the same across countries [23]. The different criteria used in defining urban areas in 14 countries included in the study are given in additional information (Additional file 1: Table S2).

More standardized, routinely available urbanization classifications were extracted from two global datasets: Global Rural Urban Mapping Project Urban extents (GRUMP UE) and Moderate Resolution Imaging Spectrometer (MODIS) urban extents. GRUMP UE is a freely available dataset from the Centre for International Earth Science Information Network at 1 km spatial resolution [13] derived from NOAA’s night-time lights dataset [24, 25] combined with settlements data. Urban extents are defined as contiguous lit cells from the night-time Lights with total population greater than 5000 persons or approximated based on buffered settlement points [26, 27]. MODIS urban extents on the other hand are derived from supervised classification of MODIS satellite imagery data. Contiguous cells with >50% in the built-up class covering an area greater than 1 km^2^ are defined as urban. In a study comparing eight existing urban area maps, MODIS urban extents were found to be the most accurate global urban dataset [15]. The gridded dataset is freely available to the public at 500 m resolution [28]. A fourth urbanization definition derived from a modification of the GRUMP UE to include a peri-urban class defined by assigning a true urban core with population density ≥1000 people per km^2^ while the surrounding urban extents with population <1000 defined as peri-urban areas. Population densities were derived from Gridded Population of the World version 3 (GPW3) projected to 2007. In a previous study, GRUMP UE and the GRUMP-modified UE were shown to be the best to use for malaria mapping [14]. A map comparing the four urbanization classifications used in this study is provided in Additional file 1.

### Population density

The principal source of human population distribution data used to assess the effects of population density on malaria parasite prevalence was the WorldPop surface [29]. The WorldPop dataset provides Africa-wide gridded population distribution estimates at 100 m spatial resolution projected to 2010 [30]. The WorldPop dataset re-sampled to 1 km spatial resolution and projected to each year between 2006 and 2010 was used to extract population density for each cluster in the household survey dataset in their respective year of survey. Population density extraction was done using the spatial analyst tool in ArcGIS 10 (ESRI, USA). To account for household dispersal within a cluster, binomial interpolation technique was used to calculate the average population density within 5 km of the cluster centre.

### Statistical modelling: BRT model building

Boosted regression trees (BRT) modelling was used to assess the impact of urbanization and population density on malaria prevalence adjusting for the effect of confounding factors. Boosted regression trees (BRT) is a relatively novel, but increasingly important, method of event distribution modelling in ecology and epidemiology. BRT modelling is increasingly being used in spatial modelling and was found very efficient in predicting species’ spatial distributions based on a set of environmental variables [31–33]. It has been used to produce soil predictive maps [34], remote sensing applications in land cover classification [28] and recently modelling land cover change [35].

Four separate BRT models (Models I–IV) were first constructed using each of the four urbanization classifications described and a set of common independent variables (Table 1). The main outcome was the probability of a child being malaria parasite positive. Random effects between the fourteen countries were controlled for in the model as a factor with unordered levels. A detailed description of BRT model parameterization and optimization are provided in Additional file 1. A fifth BRT model (Model V) was constructed with same set of common independent variables with population density replacing urbanization as the main explanatory variable to determine the impact of population density on falciparum malaria prevalence.

**Table 1:** Summary of the average contributions of predictor variables using a boosted regression trees (Model VII) developed with cross-validation over 25 bootstraps

Predictor	Relative contribution (%)
Child specific predictors	
Age in months	5.01 (SD 0.25)
Gender	0.38 (SD 0.05)
Malaria testing method	1.29 (SD 0.24)
Slept under a net	0.47 (SD 0.08)
Slept under ITN	0.59 (SD 0.09)
Fever in last 2 weeks	1.37 (SD 0.1)
Fever treatment	1.82 (SD 0.17)
Treatment with anti-malarial	0.43 (SD 0.06)
Mothers age in years	6.33 (SD 0.23)
Mothers education level	1.38 (SD 0.15)
Household level predictors	
HH with IRS	1.47 (SD 0.15)
Per capita net ownership	0.64 (SD 0.11)
Wealth index	3.43 (SD 0.31)
Cluster level predictors	
Population density	9.55 (SD 0.46)
CSO urban	1.5 (SD 0.2)
Enhanced vegetation index (EVI)	4.18 (SD 0.2)
Annual mean temperature	16.68 (SD 0.64)
Temperature suitability index (TSI)	5.15 (SD 0.43)
Annual mean precipitation	14.19 (SD 0.37)
Malaria seasonality (>60%)	0.15 (SD 0.03)

Between-country effects controlled for in the model accounts for 24% not represented in the table. The effect of country-specific factors not collected in DHS/MICS datasets not accounted for in the BRT models. Results shown for Model VII that includes population density, urbanization and a common set of confounding variables

For comparison purposes, a sixth BRT model (Model VI) was constructed using only the set of common independent variables excluding both urbanization and population density. Finally, a seventh BRT model (Model VII) was constructed including population density and urbanization and the common set of independent variables. The models are summarized as follows:

i. Model I: Common set of covariates (Table 1) + GRUMP UE
ii. Model II: Common set of covariates (Table 1) + CSO urban
iii. Model III: Common set of covariates (Table 1) + modified GRUMP UE
iv. Model IV: Common set of covariates (Table 1) + MODIS urban
v. Model V: Common set of covariates (Table 1) + population density
vi. Model VI: Common set of covariates (Table 1)
vii. Model VII: Common set of covariates (Table 1) + population density + CSO urban

Partial dependence plots were used to examine the effect of each predictor variable on the response (malaria positivity) after accounting for the average effect of all other variables in the model. Cross-validation techniques were used to evaluate model predictive performance, by randomly separating the dataset into a modelling dataset that was used to fit the model and a testing dataset that was excluded from model fitting and was used for testing the model’s predictive performance. The ratio model set was 50%, which defined the percentage of the data sampled at every run without replacement. This was further improved using bootstrapping techniques with 25 iterations for each of the defined BRT models (Models I–VII) and the predictive power of each model measured using the AUC. To assess which BRT model best predicted the outcome, AUC values were compared and the significance of the differences examined using a Tukey’s honest significant difference test. All BRT models were developed using the R package ‘gbm’ version 1.6–3.2 [36] and the additional functions provided in [37]. All analyses were conducted using R (version 2.15.3).

## Results

78,882 records of children aged less than 5 years tested for malaria in close to 6000 clusters were assembled for analysis from 19 household surveys across 14 African countries undertaken between 2007 and 2012. 1110 (0.01%) of the available child records had no geographic positions and were excluded. Of the remaining 77,772 child-records, 17% were positive for *P. falciparum* malaria where the majority (81%) had been tested for malaria using microscopy. A summary of the characteristics of the child-level and household level predictors that were used in the analysis is given in additional information (Additional file 1: Table S3).

**Fig. 1:**
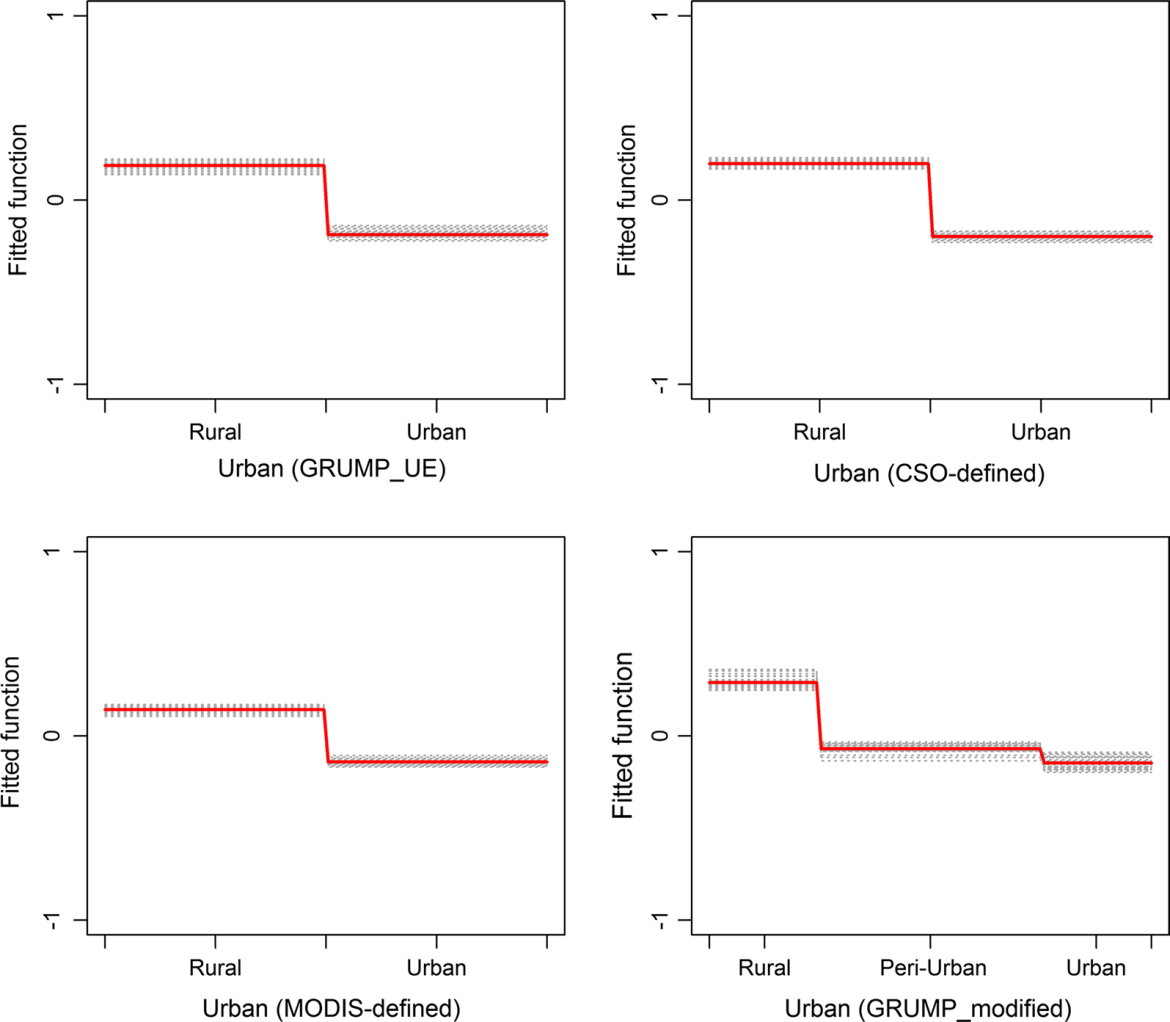
Partial dependence plot showing the relationship between urbanization and the response, malaria positivity. After accounting for the average effect of other explanatory variables in Models I, II, III and IV. Effect after accounting for the average effect of other explanatory variables. Children living in urban areas were associated with a lower risk of malaria infection compared to children in rural areas. *Y axis* is on the logit scale and is centred to have zero mean over the data distribution. *Dashes* at inside top of plots show the data distribution of predictor variables in deciles. Results for each of the 25 bootstrap runs are shown in *black dashed lines* while the *red line* represents the average/mean plot

**Fig. 2:**
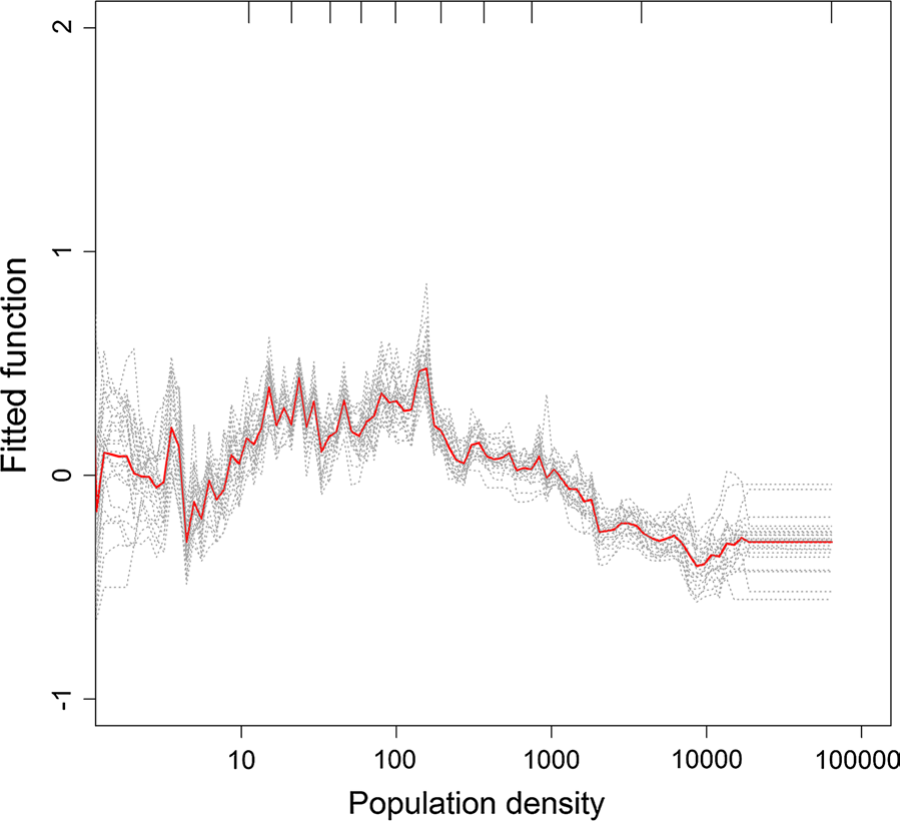
Partial dependence plot showing the relationship between population density and malaria positivity. Effect after accounting for the average effect of other explanatory variables in Model V Increase in population density was associated with increasing malaria risk until a density of about 100 persons per km^2^, but a significant decrease is observed for population densities greater than 1000 persons per km^2^. Population density was transformed on the logarithmic scale due to its skewed distribution in the data. *Y axis* is on the logit scale and is centred to have zero mean over the data distribution. *Dashes* at inside top of plots show the data distribution of predictor variables in deciles. Results for each of the 25 bootstrap runs are shown in *black dashed lines* while the *red line* represents the average/mean plot

The relationship between the outcome (malaria positivity) and the main predictor variables (urbanization and population density) is shown in Figs. 1 and 2 while the relative contribution of each predictor variables on the outcome (malaria positivity) is summarized in Table 1. In the four separate BRT models constructed to evaluate which of four urbanization datasets best predicted malaria risk, urbanization was found to have an impact on malaria risk with individuals residing in urban areas shown to have decreased malaria risk compared to individuals living in rural or peri-urban areas (Fig. 1). However, no significant difference was observed between the four urbanization models using a Tukey’s honest significant difference test (p value >0.05).

In a separate model (Model V), the impact of population density on malaria infection in childhood was examined. Population density was found to be an important predictor of malaria risk with a relative contribution of 10% (Table 1). The non-linear relationship between population density and the response is shown in Fig. 2. Decreased malaria risk was observed in areas of low population (less than 10 persons per km^2^). The malaria risk curve rises with increase in population but a significant decrease is observed for population densities greater than 1000 persons per km^2^. Mean AUC value of the BRT model with population density (Model V) was higher compared to the other four models with different urbanization definitions (Models I, II, III and IV), a result that was found significant using a Tukey’s honest significant difference test (p value <0.001).

**Fig. 3:**
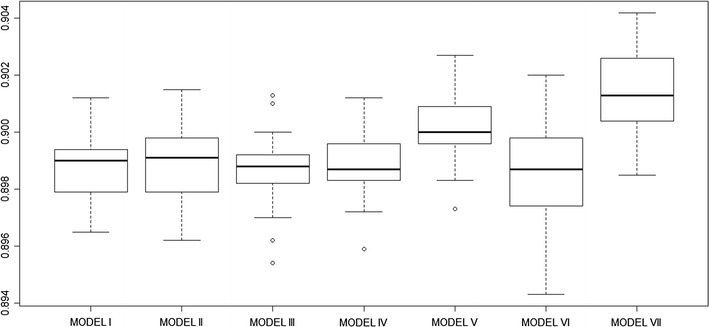
Box plot comparing accuracy assessment statistic AUC after 25 bootstrap runs for seven BRT models constructed in the analysis. AUC comparison for each of the four main models with Urbanization classification derived from GRUMP UE (Model I), CSO urban (Model II), Modified GRUMP UE (Model III) and MODIS urban (Model IV). The AUC values are then compared to values obtained from models that included population density (Model V). Model VI only includes the common set of confounding variables used in the other models and excludes any urban classification and population density while Model VII includes both the population density and urbanization (CSO urban)

Figure 3 shows box plots comparing the performance of the BRT models including population density compared to the four BRT models including urban classifications. For comparison, Model VI and Model VII were constructed to examine the impact of combining/excluding urbanization and population density in malaria prevalence models. Model VI constructed with the common set of variables excluding urbanization and population density resulted in the lowest mean AUC thus least accurate in predicting malaria positivity compared to the other models (Fig. 3) and this difference was found to be significant (Tukey test p value <0.05). On the other hand, Model VII which included both urbanization and population density performed significantly better (Tukey test p value <0.05) than all the other models (Fig. 3). The partial dependence plots of the common set of covariates controlled for in the models are given in Additional file 1.

## Discussion

Children living in urban areas were found to have a decreased risk of malaria infection compared to children residing in rural areas in all the four urbanization datasets used: CSO urban, GRUMP UE, modified GRUMP UE, MODIS urban (Fig. 1). Malaria risk was shown to decline from rural areas through peri-urban settlements to urban central areas using the modified GRUMP UE dataset. These are not new findings and are consistent with previous studies comparing malaria risk according to settlement patterns [2, 3, 11, 12, 38]. In this study however, the modelling procedure used takes into account the average effects of other variables influencing malaria risk. In addition, the BRT models controlled for the interaction between variables and thus was able to tease out the true effects of urbanization on malaria risk.

In general, this study shows that urbanization is an important predictor of malaria risk. The inclusion of urbanization, regardless of definition, significantly improved the predictive performance of all models with average AUC values increasing from 0.75 for the baseline model (Model VI) to more than 0.89 in all models including urbanization (Model I, II, III and IV). Although CSO-defined urbanization had a higher AUC, the difference in predictive performance observed amongst the four urbanization models definitions was not statistically significant.

This study also examines the relationship between human population density and malaria infection risk in children aged less than 5 years. Population density was found to be the third most important predictor of malaria infection risk with an average overall relative contribution of close to 10% to the BRT model. As seen in Fig. 2, malaria transmission is sustained as the density of human hosts increases but a decline in risk is observed in densely populated areas with densities greater than 1000 persons per km^2^. Previous studies describing malaria transmission have linked high human population densities in urban areas to low entomological inoculation rates (EIR) by reducing the overall chances of a host getting an infective bite when vector densities are low [5, 6].

Replacing urbanization with population density significantly improved the predictive performance of the BRT model (Fig. 3). The BRT model with population density was found to perform significantly better (p values <0.001) in predicting malaria risk when I compared the model’s predictive performance to the other four models with urbanization classification (Fig. 3). Given the challenges in uniformly classifying urban areas across different countries, population density may be a more accurate and reliable metric to adjust for the patterns of malaria risk in densely populated urban areas. Malaria transmission has been shown to progressively decrease from rural to peri-urban areas and from peri-urban areas to urban centre in a review of studies on malaria transmission [3]. With population density, it is also possible to more realistically model the progressive transition in malaria risk from densely populated urban centres to surrounding less densely populated peri-urban areas and on to rural areas. This provides an advantage over urbanization classifications that tend to force artificial limits in malaria risk between urban and rural area most often missing the transition in risk. Previous studies describing malaria transmission have used a single rule to “force down” infection risks in all urban and peri-urban regardless of their location or characteristics of the urban extent [1, 8, 10, 39–41]. However, malaria risk is not uniform between or within urban extents and can vary significantly within a city [2, 42–45]. Including population density in malaria modelling is important because the risk of malaria can be different in high-density cities and low-density cities, yet both would be classified as “urban”.

While previous efforts to map the intensity of malaria transmission in Africa have used only urban classification to “force down” infection risks in urban areas, this study has shown that including both urbanization and population density in the BRT model had a more significant impact in predicting malaria prevalence. Model VI which excludes both urbanization and population density performed poorly compared to other models that included either population density or urbanization. The use of population density contributes significantly to the models’ predictive performance compared to any of the previous models that included urbanization only. This implies that there are aspects of the influence of urbanization on malaria prevalence that cannot be solely explained by high population density or population distribution patterns with the final model (Model VII) that includes both urbanization and population density resulting in the best performing model (Fig. 3) compared to other models that included either or both population density and urbanization.

There are however some limitations to this study. Although the WorldPop dataset used in this study provides the best continent wide estimate of population distribution, it is prone to some under/overestimation as it relies on national census data, which is conducted in varying years for different countries and conducted at wide intervals (10 years or more in some countries). Population estimates for any year between censuses were projected using population growth estimates from the previous census period, which may not always be precise. In addition, the frequency of DHS/MICS household surveys was not sufficient to account for annual variation in malaria risk and thus constant variance was assumed for the period of study. There are also some limitations associated with environmental datasets used in this study. In order to account for the effect of time on environmental determinants of malaria, the environmental covariates used must be matched with the observed data on malaria transmission. However, the environmental covariates are rarely available at time points that correspond with the date of surveys as most are derived from long-term processed remotely sensed satellite imagery or modelled climatic data generated as synoptic estimates that do not represent a specific year [38, 46]. In an ideal experiment, individual-level malaria positivity data with geographic coordinates are household level would be linked to up to date urbanization and population density datasets that are comparable across countries with the effect of environmental variables controlled for using high resolution environmental datasets that can be matched to the period of household survey. In extracting environmental variables, additional bias could be introduced by using a radius around a cluster radius. This is however inherent when using DHS datasets where surveys with spatial information, geographic coordinates are provided for the sampling clusters, consisting of 15–30 households spread over up to 5 km in rural areas and 2 km or less in urban areas [19]. The level of bias introduced can be minimized by using household geographic coordinates to relate household members’ malaria positivity to environmental conditions obtained from equally high resolution environmental datasets. The use of household coordinates may however be limited in surveys reporting HIV due to confidentiality issues.

Additionally, dichotomous classification doesn’t always effectively describe urbanization. The degree of urbanization can be defined based demographic characteristics like population density, economic activities, infrastructure, social and cultural behaviours or a combination of these [23, 47, 48]. Demographic characteristics can be combined with satellite-derived spatial datasets to better describe urbanization as a continuum that incorporated peri-urban growth. In this study, population density was combined with satellite derived urban extents to define peri-urban areas in Model V (Table 1). The application of population density in replacing dichotomous urban classifications in modelling malaria transmission patterns was also evaluated. Population density was shown to more realistically model the continuum transition from densely populated urban centres to surrounding less densely populated peri-urban and rural areas in relation to malaria risk. There is also potential to use other demographic indicators linked to functional definitions of urbanization, often measuring level of poverty and urban-ness, like household electrification, access to improved drinking water and toilet facilities as well as housing quality [49] to describe the urbanization continuum. However, there still remains a challenge in a universal definition to quantify urbanization with little consensus among national governments and international agencies making comparisons and aggregation across countries difficult [48, 49]. There are on-going efforts to globally map settlement extents at higher resolutions of 10–30 m [50–53]. However, these projects are very recent and still in developing/testing phase so could not be used in this study. The availability of these datasets that reflect the current extents of rapidly growing urban areas especially in Africa can potentially improve the estimation of the impact of urbanization on malaria risk.

## Conclusion

In general, this study shows urbanization and human population density influence the predicted risk of malaria infection. This study shows that the risk of infection with malaria does exist in these densely populated urban areas across Africa. Despite a reduction in malaria risk associated with increasing population density, these high-density settlement areas do not have zero risks of infection. As such it is important to recognize that the public health burdens in some settings might be large and significant. Africa’s population is projected to double to close to 2 billion over the next 35 years with most of this growth expected to be concentrated in urban areas. Future malaria risk models can, therefore, be improved by including both population density and urbanization, which have both been shown to have significant impact on malaria risk in this study. Population density is more interpretable while urbanization would account for aspects of urban life not simply reflected by population density.

## Additional file

**Additional file 1.** Summary of records from household data survey datasets used and criteria used to define urban areas at national level in 14 African countries.
