# Supplementary material for: The impact of urbanization and population density on childhood Plasmodium falciparum parasite prevalence rates in Africa
Source: Malar J. 2017 Jan 26;16:49. doi: 10.1186/s12936-017-1694-2 (PMC5270336; doi:10.1186/s12936-017-1694-2)
Supplement: Supplementary file 1 — Additional file 1. Summary of records from household data survey datasets used and criteria used to define urban areas at national level in 14 African countries. [file 12936_2017_1694_MOESM1_ESM.docx]

## Supplementary Information

### Data assembly

Table S1: Summary of records from household data survey datasets used.

| **Country** | | **Surveys Type & Year** | | **No of Clusters** | **No of Households** |
| --- | --- | --- | --- | --- | --- |
| Angola | | MIS 2007 | | 115 | 1513 |
|  | | MIS 2011 | | 228 | 1962 |
| Burkina Faso | | DHS 2010 | | 540 | 3811 |
| Cameroon | | DHS 2011 | | 574 | 3426 |
| Cote d'Ivoire | | DHS 2011 | | 341 | 2214 |
| Djibouti | | MIS 2008-2009 | | 132 | 925 |
| Kenya | | MIS 2010 | | 241 | 5787 |
| Liberia | | MIS 2011 | | 150 | 1211 |
|  | | MIS 2009 | | 266 | 4355 |
| Madagascar | | MIS 2011 | | 140 | 1626 |
| Malawi | | DHS 2010 | | 106 | 1011 |
| Mali | | MIS 2010 | | 239 | 3043 |
| Nigeria | | DHS 2007 | | 246 | 3521 |
| Rwanda | | DHS 2010 | | 492 | 3088 |
|  | | DHS 2010-2011 | | 389 | 1945 |
| Senegal | | MIS 2008 | | 315 | 1999 |
|  | | DHS 2010 - 2011 | | 497 | 4239 |
| Tanzania | | AIS*/MIS 2007 | | 465 | 4283 |
|  | | MIS 2007 | | 115 | 1513 |
| **Totals** | |  | |  |  |
| Total number of Children Tested for Malaria | |  | | 77772 |  |
| Total number of Households | |  | | 51472 |  |
| Total number of Clusters | |  | | 5591 |  |

***Footnote**: AIDS Indicator Survey (AIS) developed primarily to obtain indicators for monitoring of national HIV/AIDS programs and also include modules that test for malaria [www.dhsprogram.com]. Swaziland MIS 2010 not included in the study as this country is on a malaria elimination pathway and the only infections detected were suspected imported cases [MoHSW, 2010].

**Table S2:** Criteria used to define urban areas at national level in 14 African countries

(Adapted from World Urbanization prospects: 2011 revision; UN, 2012).

| **Country** | **Urban definition** |
| --- | --- |
| Angola | Localities with a population of 2,000 or more. |
| Burkina Faso | Localities with 10,000 inhabitants or more and with sufficient socio-economic and administrative infrastructures. |
| Cameroon | Administrative centres of territorial units (district, sub-division, division or province) or/and any locality with more than 5,000 inhabitants and with sufficient socio-economic and administrative infrastructures. |
| Côte d'Ivoire | Urban agglomerations containing more than 10,000 inhabitants; agglomerations with populations ranging from 4,000 to 10,000 persons with more than 50 per cent of the households engaged in non-agricultural activities; and the administrative centres of Grand Lahoun and Dabakala. Excludes the milieu urbane of Bouna, which has a population of 11,000 persons. |
| Djibouti | Localities of 1,500 inhabitants or more. |
| Kenya | Municipalities, town councils, and other urban centres with 2,000 inhabitants or more. |
| Liberia | Localities with 2,000 inhabitants or more. |
| Madagascar | Centres with 5,000 inhabitants or more. |
| Malawi | All townships, town planning areas and district centres. |
| Mali | Up to 1987 census, localities with 5,000 inhabitants or more and district centres. Due to several historical changes in definition of urban areas, urban is defined in this publication as localities with 30,000 inhabitants or more in 1998 census, and with 40,000 inhabitants or more in 2009 census. |
| Nigeria | Towns with 20,000 inhabitants or more whose occupations are not mainly agrarian. |
| Rwanda | Administrative centres of préfectures and important agglomerations and their surroundings. |
| Senegal | Agglomerations of 10,000 inhabitants or more. |
| United Republic of Tanzania | All regional and district headquarters, as well as all wards with urban characteristics (i.e., exceeding certain minimal level of size-density criteria and/or with many of their inhabitants in non-agricultural occupations). No specific numerical values of size and density are identified, and wards are defined as urban based on the decision of the District/Regional Census Committees. |

Figure S1: Map comparing the limits of urban extents as defined by (a) GRUMP (b) MODIS (C) GRUMP modified (d) CSO-defined urban clusters in the cities of Lagos, Nigeria and Dar es Salaam, Tanzania.

| **LAGOS** | 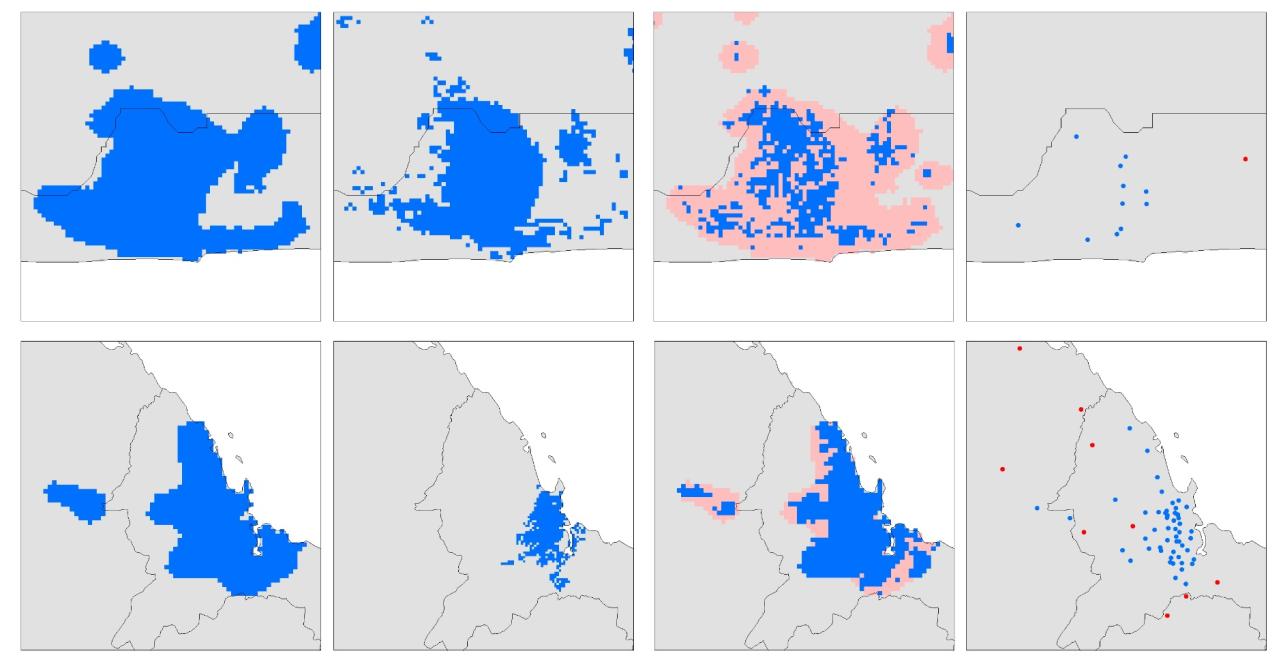 |
| --- | --- |
| **DAR ES SALAAM** |  |

(a) (b) (c) (d)

**Footnote:** Urban areas are show in blue polygons while pink shows peri-urban areas. Blue dots indicate CSO-urban clusters while red dots show CSO-rural clusters.

### Methods: BRT model optimization

To determine the optimal settings for the BRT models, the following parameters must be considered: Number of trees (nt); tree complexity (TC) and learning rate (LR). The learning rate is used to shrink the contribution of each tree as it is added to the model. Decreasing LR increases the number of trees required. Generally, smaller LR values are preferred although these increase computation time especially as the number of observations increase. TC is the number of tree nodes and represents the level of interactions between variables. Ultimately, the learning rate and tree complexity combined determine the total number of trees in the final model. In fitting an optimal BRT model, the aim is to find the combination of parameters (LR, TC and nt) that achieves minimum predictive error (minimum error for predictions to independent samples) while keeping reasonable computation times.

For this study, four combinations of parameters were explored. Three models with the learning rate (LR) set at 0.005, 0.01 and 0.05 respectively were fitted with a tree complexity of 5 set for the three models. A fourth model was fitted with the tree complexity changed to 1 and the learning rate (LR) set to 0.01. A bag fraction of 0.75 was set for all the models, meaning that at each iteration, 75% of the data are drawn at random, without replacement, from the full training set. The first two models with a low LR (0.005 & 0.01) required thousands of trees to reach minimum error and terminated when the maximum tree limit was reached without achieving minimum error. The third model with a faster learning rate at 0.05 and tree complexity of 5 resulted in a mean residual deviance of 0.423 fitted using 3850 trees. The fourth model resulted in a higher mean residual deviance of 0.641 fitted using 7500 trees. The third model achieved the best predictive performance because it had the lowest mean residual deviance recorded and thus the parameters for this model (LR = 0.05, TC = 5) were selected for subsequent BRT models for the study. Figure S2 shows the relationship between number of trees and mean residual deviance in this third model. The trend indicates that as the number of trees increases, the mean residual deviance decreases with the model terminating when the lowest mean residual deviance was achieved and the maximum number of trees that were fitted recorded. The optimal number of trees *nt* was determined using the gbm.step function provided by [Elith *et al.,* 2008]. BRT models were developed using the R package ‘gbm’ version 1.6-3.2 [Ridgeway, 2009] and the additional functions provided in Elith *et al.* (2008). All analyses were conducted using RStudio (version 0.97.551 – R Development Core Team, http://www.r-project.org).

Figure S2: BRT optimization plot showing minimization of model deviance with the stage-wise addition of trees.

Plot shown for the third BRT model fitted with learning rate = 0.05 and Tree complexity = 5. The green vertical line indicates the maximum number of trees (3850) that were fitted to achieve the lowest mean residual deviance of 0.423 shown by the red horizontal line.


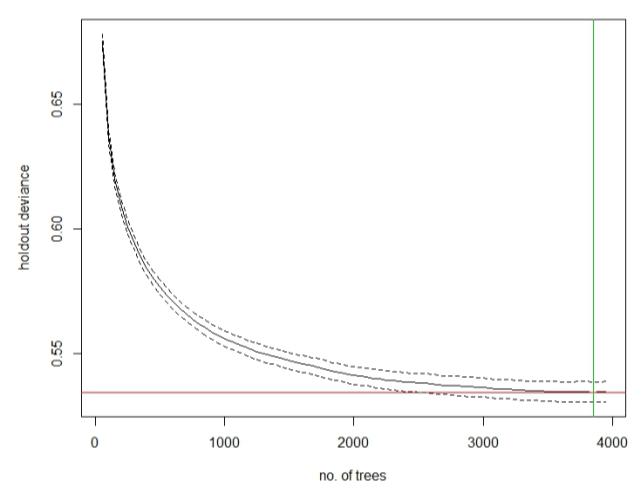


### Results

Table S3: Descriptive statistics of child-level, household-level and cluster level predictors used for analysis.

| **Child specific predictors** | |  | |  |  | |  |
| --- | --- | --- | --- | --- | --- | --- | --- |
|  | | No | | Yes (%) |  | |  |
| Malaria positive | | 64416 | | 13356 (17.2) |  | |  |
| Gender (Male) | | 39114 | | 38658 (49.7) |  | |  |
| Malaria Testing Method (Microscopy) | | 14488 | | 63284 (81.2) |  | |  |
| Slept under net | | 39870 | | 37902 (48.7) |  | |  |
| Slept under ITN | | 43324 | | 34448 (44.3) |  | |  |
| Fever in last two weeks^1^ | | 29607 | | 13486 (31.3) |  | |  |
| Fever Today^3^ | | 1721 | | 426 (19.8) |  | |  |
| Fever Treatment^1,2^ | | 10115 | | 9321 (48) |  | |  |
| Treatment with anti-malarial^1,2^ | | 7548 | | 3424 (31.2) |  | |  |
| Mothers Education Level^4^ | |  | |  |  | |  |
| None | |  | | 26741 (34.4) |  | |  |
| Primary | |  | | 28816 (37.1) |  | |  |
| Secondary | |  | | 8378 (10.8) |  | |  |
| Tertiary or Higher | |  | | 902 (1.2) |  | |  |
| Missing | |  | | 12935 (16.6) |  | |  |
|  | | Mean | | Min | Max | |  |
| Age in months | | 32 | | 0 | 59 | |  |
| Mothers Age in Years | | 29.4 | | 15 | 49 | |  |
| **Household level predictors** |  | |  | | |  | |
|  | No | | Yes (%) | | |  | |
| Children in HH with IRS^5^ | 54140 | | 9266 (14.6) | | |  | |
|  | Mean | | Min | | | Max | |
| Number of Nets per HH^6^ | 1.83 | | 0 | | | 29 | |
| Number of People/HH^6^ | 7.93 | | 2 | | | 61 | |
| Per Capita net ownership^6^ | 0.15 | | 0 | | | 2 | |
| Wealth Index^7^ |  | |  | | |  | |
| 1 (Poorest) |  | | 16376 (21.1) | | |  | |
| 2 |  | | 15682 (20.2) | | |  | |
| 3 |  | | 14234 (18.3) | | |  | |
| 4 |  | | 13142 (16.9) | | |  | |
| 5 (least poor) |  | | 10863 (14.0) | | |  | |
| Missing |  | | 7475 (9.6) | | |  | |
| **Cluster level predictors** |  | |  | | |  | |
| Urbanization | Rural | | Urban | | | Peri-urban | |
| CSO urban | 58065 | | 19707 (25.3) | | |  | |
| GRUMP UE | 62557 | | 15215 (19.6) | | |  | |
| Modified GRUMP UE | 62526 | | 7579 (9.8) | | | 7667 | |
| MODIS urban | 71219 | | 6553 (8.4) | | |  | |

**Table S3 (continued):** Descriptive statistics of child-level, household-level and cluster level predictors used for analysis

| **Cluster level predictors** |  |  |  |
| --- | --- | --- | --- |
| **Number of children living in areas with** | |  | **Total (%)** |
| Population density >1000 persons per km^8^ | | 13494 (1.75) | |
| EVI-determined arid area^9^ | | 1295 (0.17) | |
| Annual mean temperature less than 16⁰ C ^10^ | | 1427 (0.18) | |
| TSI-determined malaria unsuitable areas^11^ | | 4282 (0.55) | |
| Annual mean precipitation less than 50mm^12^ | | 11521 (1.49) | |
| Seasonal malaria | | 24192 (3.13) | |

**Footnote to Table 3.7:**

1 - Information on if child had experience fever , treated for fever or given anti-malarial drugs in the previous 14 days was not assembled in Angola MIS 2007, Cameroon DHS 2011, Cote d’Ivoire DHS 2007, Rwanda DHS 2007 & Tanzania AIS 2007 surveys.

2 - Additionally, information on treatment for fever was not given in Senegal MIS 2008 and Liberia MIS 2009 surveys

3 – Information on if child reported fever on the day of survey only reported in Djibouti MIS 2008/09, Malawi MIS 2012 and Mali DHS 2010 surveys.

4 – Caretaker’s age and education level not found in Djibouti MIS 2008/09, Mali DHS 2010, Nigeria MIS 2010 and Rwanda DHS 2007 & 2010 surveys

5 – IRS use in household information not found for Liberia MIS 2009 survey

6 – Nets per capita not calculated for Djibouti MIS 2009 & Kenya MIS 2010 surveys

7– The wealth index as measured the household surveys is a composite measure of a household's cumulative living standards. It is calculated using easy-to-collect data on a household’s ownership of selected assets, such as televisions and bicycles; materials used for housing construction; and types of water access and sanitation facilities. The wealth index is generated using principal components analysis and places individual households on a continuous scale of relative wealth. DHS separates all interviewed households into five wealth quintiles (ranging from Lowest, Second, Middle, Fourth, and highest) and can be used to compare the influence of wealth on various population, health and nutrition indicators (www.measuredhs.com/topics/Wealth-Index.cfm). Wealth Index not given in Kenya MIS 2010 & Mali DHS 2010

8 – Population density >1000 persons per km^2^ was used to define a true urban core in Tatem *et al.,* (2008).

9 – EVI values less than or equal to 0.1 (EVI <=0.1) were used to define an aridity mask. For an area to support transmission, EVI values greater than 0.1 must be recorded to at least two consecutive months in a synoptic year [Guerra *et al.*, 2008]

10 – Laboratory experiments have shown that at lower temperatures (<16^o^C) mosquito larvae were unable to produce viable adults and at high temperatures (>34^o^C) lead to almost 100% larval mortality [Bayoh & Lindsay, 2003; 2004]. No clusters fell in areas with temperatures >34^o^C.

11–Temperature suitability index (TSI developed from a biological model that accounts for the dependency of the malaria transmission cycle on temperature [Gething *et al.,* 2011].

12 – Arid & semi-arid areas defined as areas falling within the rainfall zones < 80mm monthly (< 600 mm annually) [FAO, 1987]

### Outputs of common set of covariates controlled for in the BRT models: Relationship between malaria risk and other explanatory variables:

Figure S3: Partial dependence plots showing the effect different child-level predictors had on the probability of a child testing for malaria after accounting for the average effect of other explanatory variables. Results for each of the 25 bootstrap runs are shown in black dashed lines while the red line represents the average /mean plot.

| **Fitted function** | 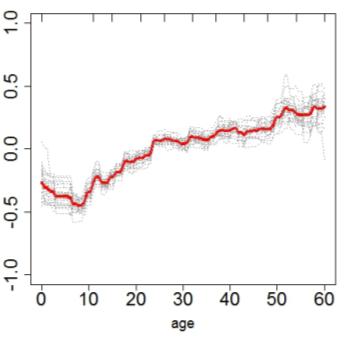 | 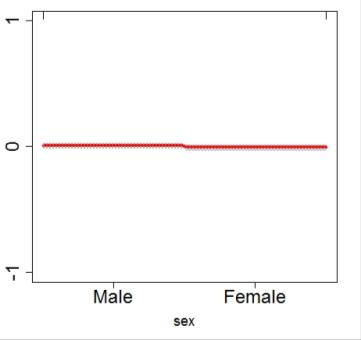 |
| --- | --- | --- |
|  | (a) | (b) |
| **Fitted function** | 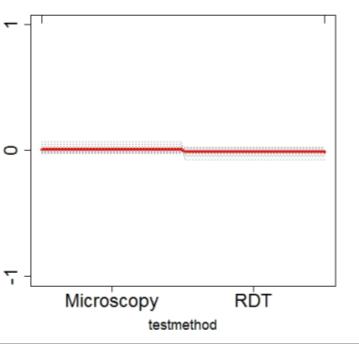 | 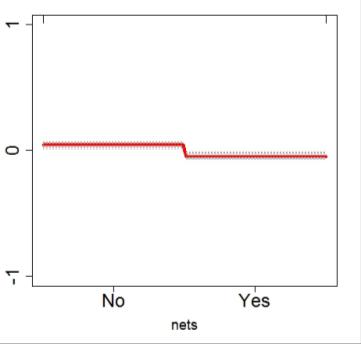 |
|  | (c) | (d) |

**Footnote**: Results are shown for (a) Age in months (b) Gender (c) Malaria testing method (d) Slept under net. Y axes are on the logit scale and are centered to have zero mean over the data distribution. Dashes at inside top of plots show the data distribution of predictor variables in deciles.

**Figure S3** (continued)

| **Fitted function** | 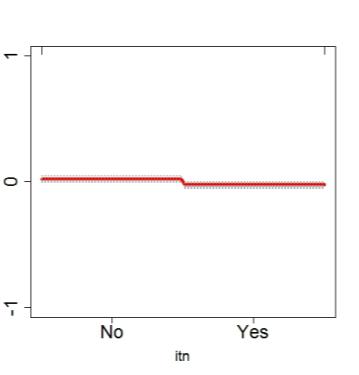 | 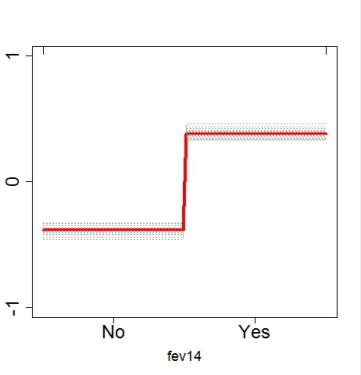 |
| --- | --- | --- |
|  | (e) | (f) |
| **Fitted function** | 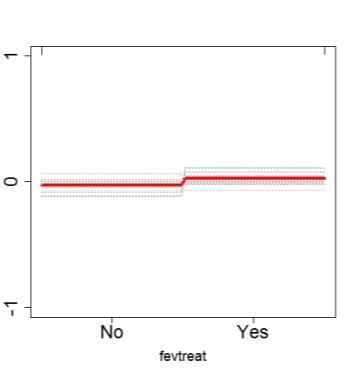 | 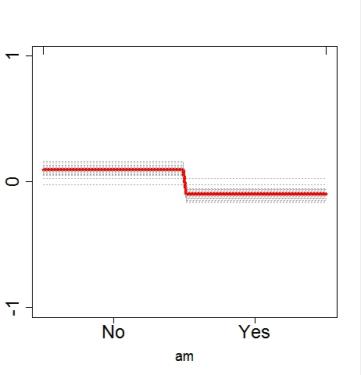 |
|  | (g) | (h) |
| **Fitted function** | 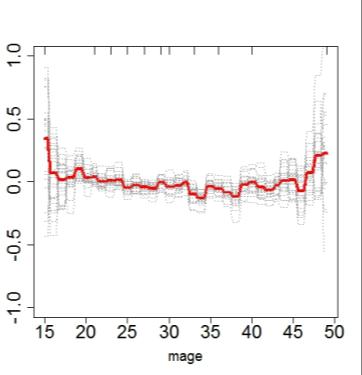 | 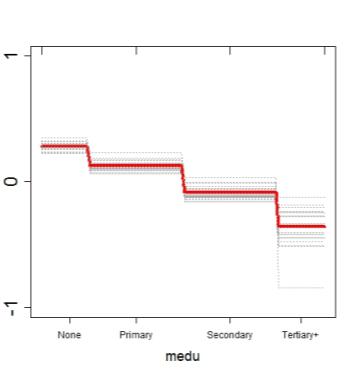 |
|  | (i) | (j) |

**Footnote**: Results are shown for (e) Slept under ITN (f) Fever in last two weeks (g) Fever treatment (h) Treatment with anti-malarial (i) Mothers age in years and (j) Mothers education level. Y axes are on the logit scale and are centered to have zero mean over the data distribution. Dashes at inside top of plots show the data distribution of predictor variables in deciles.

Figure S4: Partial dependence plots showing the effect different household level predictors had on the probability of a child testing for malaria after accounting for the average effect of other explanatory variables. Results for each of the 25 bootstrap runs are shown in black dashed lines while the red line represents the average /mean plot.

**
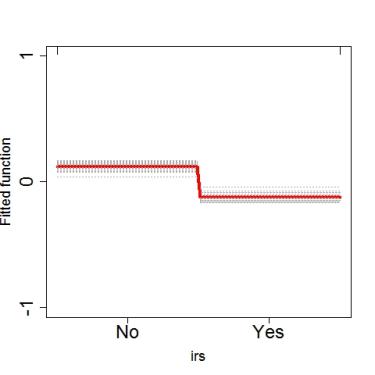

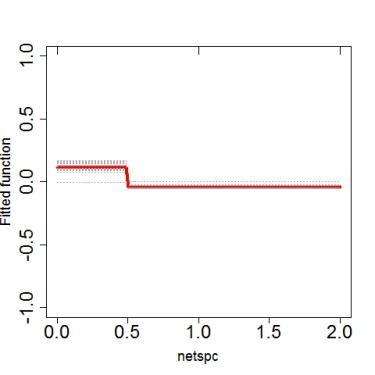
**

(a) (b)

**
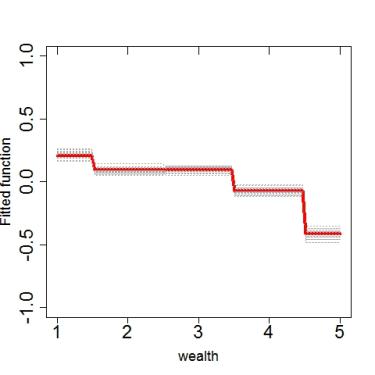
**

(c)

Footnote: Results are shown for (a) HH with IRS (b) Per capita net ownership and (c) Wealth index. Y axes are on the logit scale and are centered to have zero mean over the data distribution. Dashes at inside top of plots show the data distribution of predictor variables in deciles

**Figure S5**: Partial dependence plots showing the effect different cluster level predictors had on the probability of a child testing for malaria after accounting for the average effect of other explanatory variables. Results for each of the 25 bootstrap runs are shown in black dashed lines while the red line represents the average /mean plot.


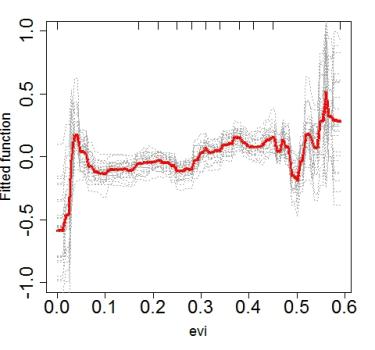

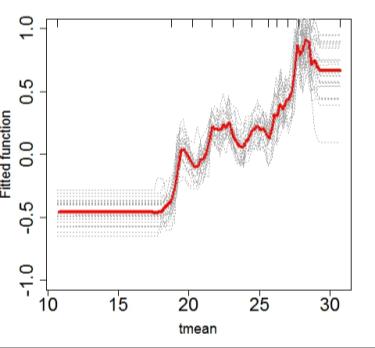


(a) (b)


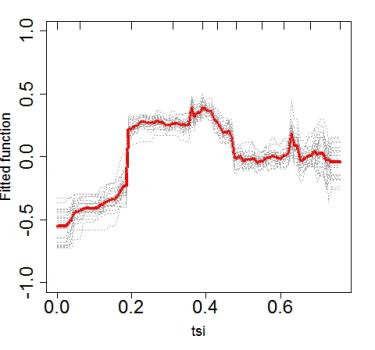

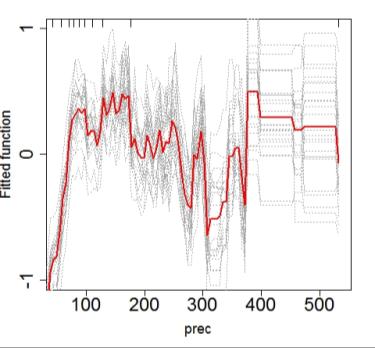


(c) (d)


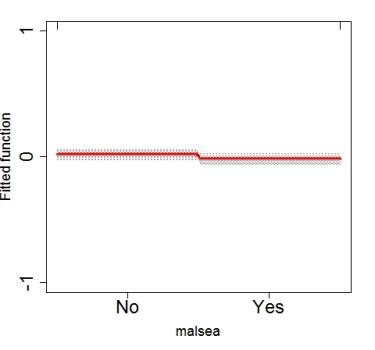


(e)

**Footnote**: Results are shown for (a) EVI (b) Annual mean temperature (c) TSI (d) Annual mean precipitation and (e) Malaria seasonality (>60%). Y axes are on the logit scale and are centered to have zero mean over the data distribution. Dashes at inside top of plots show the data distribution of predictor variables in deciles
